# Associations of Salivary BPIFA1 Protein in Chronic Periodontitis Patients with Type 2 Diabetes Mellitus

**DOI:** 10.1155/2017/1087017

**Published:** 2017-10-04

**Authors:** Yue Guo, Lin-Na Guo, Jun-Fei Zhu, Chen-Yi Tang, Yun-Zhi Feng, Hou-De Zhou

**Affiliations:** ^1^Department of Metabolism & Endocrinology, National Clinical Research Center for Metabolic Disease, The Second Xiangya Hospital, Central South University, Changsha, Hunan 410011, China; ^2^Department of Stomatology, The Second Xiangya Hospital, Central South University, Changsha, Hunan 410011, China

## Abstract

**Aims:**

To explore the differences in salivary BPI fold containing family A, member 1 (BPIFA1) concentration among type 2 diabetes mellitus (T2DM) subjects with various severities of chronic periodontitis and to determine whether BPIFA1 in saliva can be used as a potential biomarker of T2DM.

**Methods:**

Unstimulated saliva samples were collected from 44 subjects with T2DM and 44 without T2DM (NDM). Additionally, demographic data and general health parameters, including fasting blood glucose (FBG) and body mass index (BMI), were collected. We also detected full-mouth clinical periodontal parameters including probing pocket depth (PPD), clinical attachment level (CAL), bleeding index (BI), and plaque index (PLI). Salivary BPIFA1, tumor necrosis factor-*α* (TNF-*α*), and interleukin-6 (IL-6) concentrations were also detected.

**Results:**

BPIFA1 in saliva was detected at relatively high levels. T2DM subjects had decreased salivary BPIFA1 concentrations (*P* = 0.031). In T2DM subjects with nonperiodontitis or severe periodontitis, the level of BPIFA1 was significantly lower compared with that of NDM. Salivary TNF-*α* concentration displayed a similar trend to BPIFA1 in the NDM group.

**Conclusions:**

BPIFA1 protein is rich in saliva and might be used as a potential predictive biomarker of T2DM, especially in patients with severe periodontitis and nonperiodontitis. This trial is registered with ChiCTR-ROC-17010310.

## 1. Introduction

Type 2 diabetes mellitus (T2DM) is a multifactorial metabolic disease with recent evidence suggesting that it causes chronic subclinical inflammation [1], which can lead to serious complications such as kidney failure, blindness, cardiovascular disease, ulcers, and infection of the lower extremities [2]. In the United States, it has been reported that type 2 diabetes could be present for up to 9 to 12 years before initial clinical diagnosis [3]. A large-sample study conducted by the Centers for Disease Control and Prevention in 2014 estimated that 29.1 million people had diabetes and that 27.8% of these were undiagnosed [4]. Chronic periodontitis is also a chronic inflammatory disease caused by Gram-negative periodontal bacteria, which affects the supporting structures of the teeth and leads to the destruction of connective tissue and alveolar bone, with eventual tooth loss [5]. A recent study showed that the inflammation caused by chronic periodontitis could reduce glucose uptake and release of insulin, which concomitantly increased the odds of diabetes (odds ratio, OR = 1.5–2.1) [5, 6]. Diabetic patients are susceptible to periodontal diseases which could be regarded as the sixth complication of diabetes [7]. Screening patients with periodontitis can improve the diagnostic rate of T2DM [8]. Therefore, the discovery of sensitive biomarkers associated with T2DM in periodontitis individuals is clinically meaningful.

The bactericidal permeability increasing (BPI) fold containing family (*BPIF*) gene, previously termed named as the palate, lung, and nasal epithelium clone (*PLUNC*) gene [9], belongs to a multigene family located on a 300 kb locus on chromosome 20q11.2 in humans. BPIF can be divided into two subgroups. One group is the short type BPIF (BPIFA) containing only one domain of BPI protein and the another group is the long type BPIF (BPIFB) containing two domains similar to the whole BPI molecule [10]. Weston et al. found that BPIFA1 was highly expressed in the nasopharyngeal cavity [11]. Given the close anatomical relationship between the nasopharyngeal and oral cavities, it is possible that substances in the nasopharyngeal cavity could influence salivary components [12]. We also detected the distribution of the tissue distribution of BPIFA1 and found that BPIFA1 can also be detected in the oral cavity including the palate, parotid glands, and saliva [13]. Therefore, specific factors that are highly expressed in the nasal mucosa might be detected in saliva with both high sensitivity and specificity. Meanwhile, we successfully generated a polyclonal BPIFA1 antibody and found that BPIFA1 could bind to lipopolysaccharide (LPS), suggesting that BPIFA1 had antibacterial properties since it could bind bacterial LPS [13, 14]. Recently, Abdolhosseini et al. found that a synthetic peptide (GL13NH2) from the parotid secretory protein, a member of BPIF, could reduce the LPS-stimulated release of tumor necrosis factor-*α* (TNF-*α*) from the RAW 264.7 cell line, *in vitro* [15]. Moreover, our previous study also found that BPIFA1 can modulate cytokine production through its BPI domain [16]. Thus, BPIFA1 appears to be crucial in both pathogenic bacteria and human innate immune responses (Supplementary Figure 1 available online at https://doi.org/10.1155/2017/1087017).

Chronic periodontitis is caused by Gram-negative periodontal bacteria containing LPS and may lead to the changes in BPIFA1 concentration in saliva. A study concerning proteomic profiles of whole unstimulated saliva conducted by Wu et al. suggested that BPIFA2 was reduced in the saliva of patients with generalized aggressive periodontitis [17]. T2DM could also cause a systemic inflammatory state [18], which affects the expression level of salivary cytokines. Exploring salivary proteomes in edentulous patients with T2DM confirmed that the BPIFA1 expression level was lower in the edentulous diabetic group [2]. Therefore, research is required to investigate the expression level of salivary BPIFA1 in patients with chronic periodontitis with T2DM.

No studies have been reported concerning the anti-inflammatory mechanism of BPIFA1 in saliva. Ou et al. identified that BPIFA1 could reduce the inflammatory response by inhibiting the Toll-like receptor (TLR) 9/NF-*κ*B pathway, which resulted in low *in vitro* expression of IL-6, IL-8, IL-1*β*, and TNF-*α* [19]. Salivary TNF-*α* and interleukin-6 (IL-6) as inflammatory cytokines could reflect the periodontal status of the oral cavity [20, 21] and may both be related to modulation of the inflammatory response of BPIFA1. Therefore, our study aimed to appraise whether BPIFA1 in saliva could be used as a potential biomarker of T2DM and explore the association between BPIFA1, TNF-*α*, and/or IL-6 in saliva.

## 2. Materials and Methods

### 2.1. Subjects

The study sample comprised a total of 88 participants (aged 40–75 years): 44 with T2DM and 44 without T2DM (NDM). The subjects were stratified according to periodontal status into 8 equally numbered groups: T2DM without chronic periodontitis (*n* = 11); T2DM with mild chronic periodontitis (*n* = 11); T2DM with moderate chronic periodontitis (*n* = 11); T2DM with severe chronic periodontitis (*n* = 11); NDM without chronic periodontitis (*n* = 11); NDM with mild chronic periodontitis (*n* = 11); NDM with moderate chronic periodontitis (*n* = 11); and NDM with severe chronic periodontitis (*n* = 11). T2DM was diagnosed by specialist physicians at the hospital according to the criteria of the American Diabetes Association [22]. Briefly, the diagnosis of T2DM was based on one of the following criteria: fasting plasma glucose level of ≥126 mg/dL (≥7.0 mmol/L), random plasma glucose of ≥200 mg/dL (≥11.1 mmol/L), or plasma glucose of ≥200 mg/dL (≥11.1 mmol/L) after administration of 75 g oral glucose tolerance test (OGTT). The inclusion criteria were (i) being diagnosed with T2DM for more than one year, (ii) no antibiotics or steroidal and nonsteroidal anti-inflammatory medications being used during the last 3 weeks, and (iii) not treated with immunosuppressive chemotherapy, no current acute illness present, no professional periodontal treatment received during the last 6 months, and no ongoing pregnancy or lactation.

In order to test the effect of age, fasting blood glucose (FBG), and body mass index (BMI) on the concentration of salivary BPIFA1, patients were divided into an elderly group (≥60 years old) and a nonelderly group (<60 years old) [23]. They were further stratified into an elevated FBG group (≥6.1 mg/dL) and a normal FBG group (<6.1 mg/dL) [24] and an obese group (BMI > 28) and a nonobese group (BMI ≤ 28) [25].

All subjects were recruited from the Department of Stomatology and Health Management Center of the Second Xiangya Hospital of Central South University, China. The Ethical Committee of the Second Xiangya Hospital of Central South University approved the study protocol. Participation was confirmed by written informed consent, and the steps of clinical examination and sampling procedures were explained to each participant. All participants received oral and written hygiene instructions. This study was registered on the Chinese clinical trial registry (ChiCTR-ROC-17010310).

### 2.2. Clinical Periodontal Examination

A single examiner (LN G) carried out full-mouth periodontal parameters for all subjects. In order to determine the classification of periodontitis, probing pocket depth (PPD) and clinical attachment level (CAL) were recorded at six sites for each tooth (except the third molars). This included buccal-mesial, midbuccal, buccal-distal, lingual-mesial, midlingual, and lingual-distal sites. PPD and CAL were measured in millimeters using a manual periodontal probe (UNC15; Hu-Friedy, Chicago, IL, USA). PPD was the distance from the gingival margin to the bottom of the pocket, and CAL was the distance from the cemento-enamel junction to the bottom of the pocket. According to the recent case definition described by the Centers for Disease Control and Prevention in partnership with the American Academy of Periodontology [26], study subjects were diagnosed as mild (≥2 interproximal sites with CAL ≥3 mm and ≥2 interproximal sites with PPD ≥4 mm not on the same tooth or one site with PPD ≥5 mm), moderate (≥2 interproximal sites with CAL ≥4 mm not on the same tooth or ≥2 interproximal sites with PPD ≥5 mm not on the same tooth), severe periodontitis (≥2 interproximal sites with CAL ≥6 mm not on the same tooth and ≥1 interproximal site with PPD ≥5 mm), or not having periodontitis (no evidence of mild, moderate, or severe periodontitis).

In order to comprehensively evaluate periodontal status, other clinical parameters were employed, including bleeding index (BI), which reflects the inflammation of periodontal tissues. Furthermore, we also used the plaque index (PLI), that is, the percentage of sites with visible plaque. Measurements were made at the aforementioned six sites. BI was registered as 15 s after light probing. Clinical criteria for periodontal BI and PLI are described in Table 1. The number of teeth was also calculated.

### 2.3. Collection of Saliva Samples

Whole unstimulated salivary samples (WUS) were collected between 9:00 and 10:00 a.m., before periodontal examination, using standard techniques described by Navazesh [27]. Briefly, subjects refrained from eating, drinking, and using chewing gum, for at least 1 hour prior to evaluation. Samples were obtained by requesting subjects to initially gargle for 5 min, and then subjects were asked to spit saliva into sterile 50 mL centrifuge tubes for 5 min without swallowing. The tubes were cooled in ice water at all times. All samples were immediately centrifuged at 6000*g* for 20 min, at 4°C, to remove cellular debris. The supernatant was then stored at −80°C for subsequent analysis.

### 2.4. Enzyme-Linked Immunosorbent Assay (ELISA) Analyses

The supernatants were thawed on ice in the lab and used in the ELISA assays. For each sample, 100 *μ*L supernatant was used for the assays. The concentration of BPIFA1 (BIORBYT Company, Cambridge, United Kingdom), IL-6, and TNF-*α* (CUSABIO Inc., Wuhan, China) in supernatant of WUS was determined using an ELISA kit, according to the manufacturer's protocol. The concentration of cytokines (BPIFA1, IL-6, and TNF-*α*) was calculated from the colorimetric OD using a standard curve. All experiments were conducted at least 3 times, with similar results obtained.

### 2.5. Statistical Analysis

The statistical program SPSS (version 17.0; SPSS, Chicago, IL, USA) was used to analyze the data. We compared age, FBG, BMI, and number of teeth between the T2DM and NDM groups using the independent sample *t*-test. The Chi-squared test was used to compare the descriptive frequencies for age, gender, elevated fasting blood glucose, obesity, and severity of periodontitis. When we analyzed the periodontal parameters for various severities of periodontitis with or without T2DM, the distributions of periodontal parameters were skewed. Consequently, the nonparametric Kruskal-Wallis test was used to compare data between the T2DM and NDM groups and the Mann–Whitney test was applied for in-group comparisons of the T2DM or NDM groups. The distributions of salivary indicator concentrations were skewed, yet log conversion of these indicators was normally distributed. Therefore, the independent sample *t*-test was used to detect changes in salivary indicator concentrations according to the classification of age, gender, FBG, and BMI in subjects with or without T2DM. This *t*-test was also used to compare salivary indicators between the T2DM and NDM groups. When we analyzed these biomarkers in saliva according to severity of periodontitis and stratified by presence of T2DM, the analysis of variance (ANOVA) was used. We analyzed the curve correlation between the concentration of BPIFA1 (pg/mL) and periodontal parameters in individuals with or without T2DM by curve fitting. Logistic regression was calculated to evaluate the relationships between type 2 diabetes and salivary biomarkers. We selected T2DM status (1: NDM, 2: T2DM) as the dependent variable. In addition, we selected salivary BPIFA1 (1: 0–499 pg/mL, 2: 500–999 pg/mL, 3: 1000–1499 pg/mL, 4: 1500–1999 pg/mL, 5: 2000–2499 pg/mL, 6: 2500–2999 pg/mL, and 7: 3000–3500 pg/mL), salivary TNF-*α* (1: 0–199 pg/mL, 2: 200–399 pg/mL, 3: 400–599 pg/mL, and 4: >600 pg/mL), and salivary IL-6 (1: 0–19 pg/mL, 2: 20–39 pg/mL, 3: 40–59 pg/mL, and 4: >60 pg/mL) as the independent variables. Statistical significance was defined as *P* < 0.05.

## 3. Results

### 3.1. Demographic Characteristics and Clinical Measures in Subjects

The distribution of demographic data (Table 2) shows that the mean ages of the NDM and T2DM patients (both *n* = 44) were 53.7 ± 9.2 and 58.8 ± 10.4 (*P* > 0.05), respectively. The age ranges were 41–75 and 40–71 years, respectively. In subjects with T2DM, the serum levels of FBG were significantly higher than in those with NDM (*P* < 0.05). The proportion of elevated FBG was significantly higher in the T2DM group compared to the NDM group. There was no significant difference in BMI and the number of teeth between groups. Furthermore, there were no significant differences in the proportion of those who were elderly, male, obese, or by severity of periodontitis between groups.

Periodontal parameters by varying severities of periodontitis, with or without T2DM, are presented in Table 3. All parameters were skewed, and data for the median and interquartile range (IQR) are presented. For intragroup comparisons of T2DM or NDM, PPD, CAL, BI, and PLI (*P* < 0.05) were significantly different in the nonperiodontitis, mild periodontitis, moderate periodontitis, and severe periodontitis groups either independent of T2DM status. We also compared periodontal parameters between T2DM and NDM groups. However, there was no significant difference in periodontal parameters between T2DM and NDM groups (*P* > 0.05, Table 3).

### 3.2. Salivary BPIFA1 Concentration Was Not Affected by Age, Gender, FBG, and BMI but Decreased in T2DM Subjects

As shown in Table 4, salivary BPIFA1, TNF-*α*, and IL-6 concentrations in patients stratified by age, gender, FBG, and BMI were analyzed. There were no significant differences observed in patients classified by age, gender, FBG, and BMI. However, the concentration of TNF-*α* was significantly higher in females than in males (*P* = 0.033).

**Figure 1:**
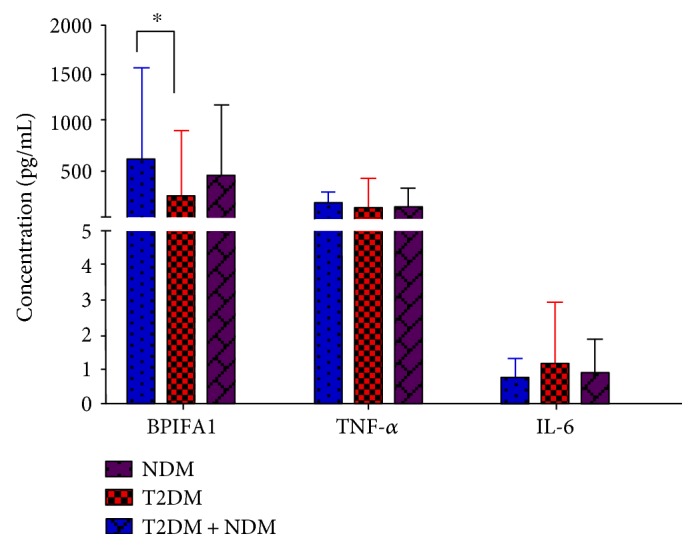
A histogram showing the concentrations of salivary BPIFA1, TNF-*α*, and IL-6 (pg/mL, median (IQR)) in NDM and T2DM subjects. The blue stripes represent NDM subjects, the red stripes represent T2DM subjects, and the purple stripes represent all subjects. Each group is presented by median (stripes) and interquartile range (bars). Salivary BPIFA1 concentration was significantly higher in the NDM group than in the T2DM group. The significant differences are indicated by an asterisk (^∗^*P* = 0.031). No significant difference in TNF-*α* or IL-6 concentration was observed between T2DM and NDM groups.

Concentrations of salivary BPIFA1, TNF-*α*, and IL-6 in subjects are shown in Figure 1. Salivary BPIFA1 concentration was significantly higher in NDM subjects compared with T2DM subjects (*P* = 0.031). There was no significant difference in the concentration of TNF-*α* and IL-6 between NDM and T2DM groups (*P* > 0.05).

### 3.3. Salivary BPIFA1 Concentrations Decreased in T2DM Individuals with Nonperiodontitis and Severe Periodontitis

**Figure 2:**
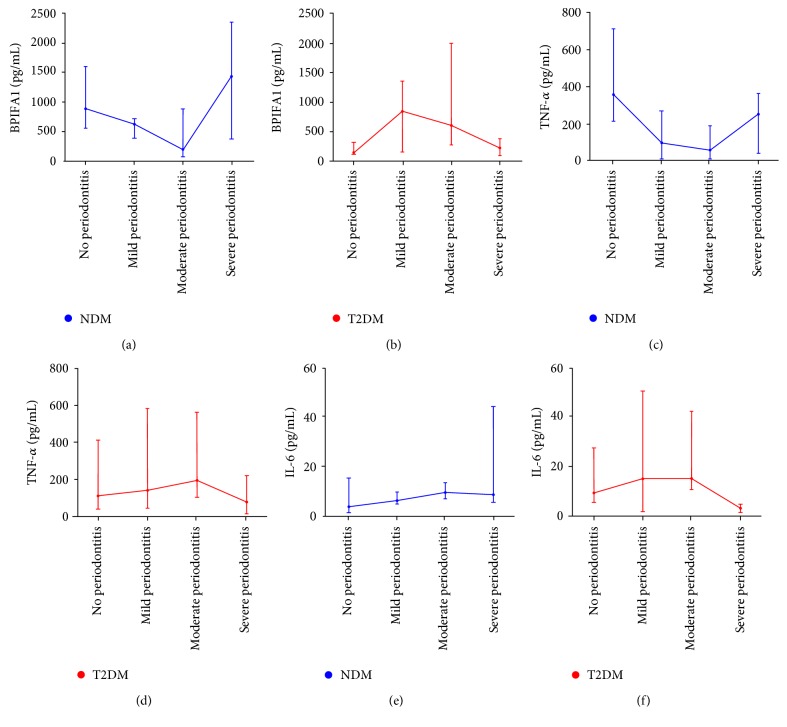
Line graph including salivary BPIFA1, TNF-*α*, and IL-6 (pg/mL, median (IQR)) concentrations in different severities of periodontitis stratified by T2DM. Blue lines and dots represent NDM subjects, and the red lines and dots represent T2DM subjects. Each group included 4 grades of periodontitis by severity including none, mild, moderate, and severe, and the median values are displayed (dots). The concentration of BPIFA1 (a) in the nonperiodontitis group was significantly higher than in the moderate periodontitis group. The level of BPIFA1 in the moderate periodontitis group was significantly lower than in the severe periodontitis group. The concentration of BPIFA1 was at its lowest in the moderate periodontitis group. However, in those with T2DM, the concentration of BPIFA1 (b) was significantly lower in the nonperiodontitis group in comparison to both the mild periodontitis and moderate periodontitis groups. BPIFA1 in the severe periodontitis group was significantly lower than in the mild and moderate periodontitis groups. Comparisons between the T2DM and NDM groups revealed that in T2DM subjects without periodontitis, BPIFA1 levels were significantly lower in those NDM subjects without periodontitis and was significantly lower in T2DM subjects with severe periodontitis individuals compared with NDM subjects with severe periodontitis. The concentration of TNF-*α* is shown in (c) and (d). In NDM subjects, the concentration of TNF-*α* in the nonperiodontitis group was significantly higher than in the mild periodontitis group and moderate periodontitis groups. BPIFA1 was significantly lower in the moderate periodontitis group compared with the severe periodontitis group. However, in those subjects with T2DM, the concentration of TNF-*α* was significantly lower in the severe periodontitis group in comparison to the moderate periodontitis group. Comparisons between T2DM and NDM groups revealed that T2DM subjects with moderate periodontitis had significantly lower BPIFA1 than NDM subjects. The concentration of IL-6 among the NDM and T2DM groups is presented in (e) and (f), which showed that the concentration of IL-6 in the moderate periodontitis group was significantly higher than in the severe periodontitis group, among T2DM subjects.

In order to compare the differences of salivary BPIFA1, TNF-*α*, and IL-6 concentrations among T2DM/NDM patients with periodontitis at different stages, we further divided the subjects into eight subgroups. In the NDM group, the concentration of BPIFA1 in those with nonperiodontitis was significantly higher than in those with moderate periodontitis (*P* = 0.019, Table 5). In the moderate periodontitis group, BPIFA1 was significantly lower than in the severe periodontitis group (*P* = 0.024). Although the concentration of BPIFA1 was lower in the moderate periodontitis group than that in the mild periodontitis group, the differences were not statistically significant (*P* > 0.05) (Table 5, Figure 2()). However, in the T2DM group, the concentration of BPIFA1 was significantly lower in those with nonperiodontitis compared to those with mild periodontitis (*P* = 0.042, Table 5 and Figure 2(b)) and moderate periodontitis (*P* = 0.005). Those with severe periodontitis had significantly lower levels of BPIFA1 than those with mild periodontitis (*P* = 0.021) and moderate periodontitis (*P* = 0.002). There was no significant differences between mild and moderate periodontitis (*P* > 0.05). When the T2DM and NDM groups were compared with each other, the level of BPIFA1 was significantly lower in the T2DM group without periodontitis (median = 110.00) compared with the NDM group without periodontitis (median = 879.89). In T2DM individuals with severe periodontitis (median = 188.05), the level of BPIFA1 was significantly lower than in those NDM individuals with severe periodontitis (median = 1441.96).

In NDM subjects, the concentration of TNF-*α* in the nonperiodontitis group was significantly higher than in the mild periodontitis group (*P* = 0.002, Table 5 and Figure 2(c)) and the moderate periodontitis group (*P* < 0.001). TNF-*α* levels were significantly lower in those with moderate periodontitis compared with those who had severe periodontitis. However, in T2DM subjects, the concentration of TNF-*α* was significantly lower in the severe periodontitis group compared with the moderate periodontitis group (*P* = 0.042, Table 5 and Figure 2(d)). Comparison between the T2DM and NDM groups revealed that the NDM group with moderate periodontitis had significantly lower levels of BPIFA1 than the T2DM group with moderate periodontitis (*P* = 0.004). The changing trend in salivary TNF-*α* concentration was similar to that of salivary BPIFA1 in those with NDM.

The concentration of IL-6 among NDM and T2DM is presented in Table 5 and Figures 2(e) and 2(f), which showed that the concentration of IL-6 in the group with moderate periodontitis was significantly higher than in the group with severe periodontitis among T2DM subjects. The changing trend of salivary IL-6 was not consistent with that of salivary BPIFA1.

### 3.4. Curve Correlations Exist between Salivary BPIFA1 Concentration and CAL in T2DM Subjects and PLI in NDM Subjects

**Figure 3:**
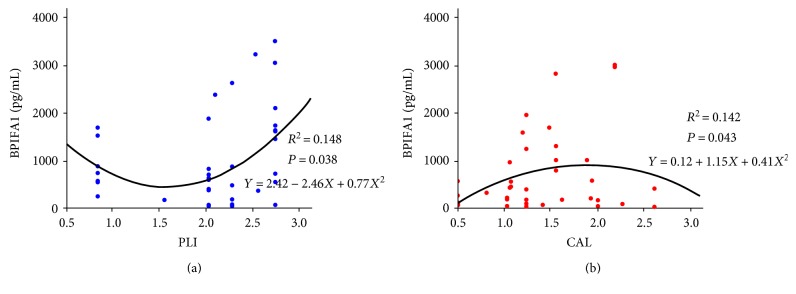
Curve correlation between the concentration of BPIFA1 (pg/mL) and periodontal parameters among individuals with or without T2DM. Blue lines and dots represent NDM subjects, and red lines and dots represent T2DM subjects. Equations and correlation coefficients are shown. As shown in (a), in NDM subjects, PLI and the concentration of salivary BPIFA1 had a statistically significant relationship in the form of a quadratic term. In T2DM subjects (b), CAL and the concentration of salivary BPIFA1 had a statistically significant relationship in the form of a quadratic term.

Using Spearman's correlation coefficients, we assessed periodontal status and concentrations of the aforementioned indicators in T2DM and NDM patients, respectively. None of Spearman's correlation coefficients approached statistical significance (*P* > 0.05, data not shown). We then analyzed the curve correlation between the concentration of BPIFA1 (pg/mL) and the periodontal parameters, PPD, CAL, BI, and PLI among individuals with or without T2DM. As shown in Figure 3, the concentration of salivary BPIFA1 correlated with PLI (*R*^2^ = 0.148, *P* = 0.038) in the form of a quadratic term, in the NDM individuals. However, in T2DM subjects, the concentration of salivary BPIFA1 was positively correlated with CAL (*R*^2^ = 0.142, *P* = 0.043) in the form of a quadratic term.

### 3.5. Salivary BPIFA1, TNF-*α*, and IL-6 Are Not Risk Indicators for T2DM

In order to explore whether salivary BPIFA1, TNF-*α*, and IL-6 were risk indicators for T2DM, logistic regression analysis was used to compare subjects with or without T2DM. As shown in Table 6, the OR of each of the salivary biomarkers was calculated but salivary BPIFA1, TNF-*α*, and IL-6 were not significantly associated with T2DM (*P* > 0.05, Table 6).

## 4. Discussion

In this study, we found that the concentration of BPIFA1 is at a relatively high level in saliva. However, expression levels of salivary BPIFA1 decreased in the T2DM group in individuals with nonperiodontitis or severe periodontitis. Therefore, we speculate that salivary BPIFA1 could be regarded as a potentially predictive biomarker of T2DM subjects especially those with severe periodontitis or nonperiodontitis. In addition, salivary BPIFA1 might reflect regulation of the inflammatory immune response in periodontitis subjects through the production of salivary TNF-*α*.

Human saliva is a rich reservoir of analytes comprising nearly 3000 proteins and 12,000 peptides [28] and is easy to obtain, while also being a noninvasive method [29]. Saliva is now regarded as a pool of biological markers and therefore has great potential for use in the prediction and diagnosis of systemic and localized diseases [30]. Many researchers have attempted to find useful biomarkers such as TNF-*α*, IL-10, IL-17, IL-12, and IL-1*β* [10, 31–33] in saliva that are associated with periodontitis to help predict or diagnose T2DM. However, low expression biomarkers are easily affected by systemic health status, which prevents these immunological markers from being widely employed [32]. As an innate immune defense molecule, BPIFA1 is highly expressed in the respiratory tract and can be detected in the oral cavity [13]. As highly expressed proteins are not easily affected by systemic disease, these proteins are more likely to be developed into biomarkers for clinical investigation. In this study, we excluded the confounding effects of age, gender, FBG, and BMI on highly expressed salivary BPIFA1. Meanwhile, due to there being no internal reference marker in saliva, we asked patients to gargle for 5 minutes before spitting out their saliva sample, to reduce systematic error in the study.

T2DM is characterized by chronic hyperglycemia which leads to protein expression changes in saliva [34]. In our study, we demonstrated that salivary BPIFA1 was significantly lower in the T2DM group compared with the NDM group (Figure 1()). Moreover, in T2DM subjects with nonperiodontitis, the concentration of BPIFA1 was significantly lower than in NDM individuals with nonperiodontitis (Table 5). The edentulous state is similar to that of nonperiodontitis because edentulism is defined as the loss of all permanent teeth, as is the terminal outcome of periodontitis and might lead to elimination of ongoing inflammation [35]. Thus, our results were consistent with the research of Border et al. who showed that the expression level of BPIFA1 was lower in diabetic edentulous subjects compared with those in nondiabetic edentulous subjects [2]. T2DM can cause systemic inflammation through overexpression of proinflammatory mediators such as IL-1, TNF-*α*, and prostaglandin E2 [36]. Therefore, our results suggest that BPIFA1 can also be affected by T2DM, as it is one of many innate immune-related proteins.

Chronic inflammation induced by *P. gingivalis* and *P. intermedia* and other bacteria in the oral cavity [5] results in periodontitis that can affect both cellular and humoral immunity, with consequent release of cytokines such as IL-2, IFN-*γ*, and TNF-*α* [7]. The release of these inflammatory cytokines can and then further lead to systemic inflammation and insulin resistance by interfering with lipid metabolism pathways [37] and ultimately promoting the development of diabetes [38]. Therefore, we divided T2DM and NDM subjects into 8 groups according to severity of periodontitis. Our results demonstrated that among T2DM subjects, the concentration of salivary BPIFA1 was significantly lower in the nonperiodontitis group compared with the mild and moderately affected groups. In subjects with severe periodontitis, the level of BPIFA1 was significantly lower than in those with moderate and mild periodontitis. This could be explained by the fact that in T2DM nonperiodontitis subjects, the body has long been in a slightly inflammatory state for a considerable period of time [18]. However, this inflammatory state might not be sufficiently inflammatory to reach the stimulation threshold of BPIFA1, which then leads to a decrease in salivary BPIFA1 concentration. In those T2DM subjects with mild and moderate stages of periodontitis, it might be that augmented systemic inflammation may be able to reach the stimulation threshold of salivary BPIFA1, which consequently leads to an increase in salivary BPIFA1 concentration through feedback mechanisms. However, in T2DM subjects with severe periodontitis, the body might become recalcitrant to BPIFA1 during inflammation. This process can be detected sensitively by BPIFA1, which is enriched in saliva. Conversely, in NDM groups, we found that the expression level of BPIFA1 was significantly higher in nonperiodontitis or severe periodontitis subjects compared with those in the moderate periodontitis group. The reason might be that during moderate periodontitis in NDM subjects, inflammation may only be slight and may not reach the stimulation threshold of BPIFA1. During severe periodontitis in NDM individuals, aggravated inflammation might be able to reach the stimulation threshold of BPIFA1, which leads to an increase in salivary BPIFA1 concentration. Bisson et al. detected the soluble form of triggering receptor on myeloid cells-1 (sTREM-1) in gingival crevicular fluid (GCF), which was a new regulator of innate immunity in periodontitis. They found that the concentration of sTREM-1 increased in severe periodontitis [39], suggesting that innate immune factors might be similarly affected in severe periodontitis. Comparisons between T2DM and NDM groups showed that the level of salivary BPIFA1 in T2DM individuals with severe periodontitis was significantly lower than that in NDM subjects with severe periodontitis, indicating that salivary BPIFA1 could be regarded as a sensitive biomarker of T2DM, especially in patients with severe periodontitis. Periodontitis can be evaluated using clinical features that include PPD, CAL, BI, and PLI [6]. We found that there was a significant curve correlation between BPIFA1 and CAL in the T2DM group, while in the NDM group, BPIFA1 significantly correlated with PLI. Other periodontal parameters were not related to the concentration of BPIFA1 (data not shown). As PLI can reflect visible plaque, which is a bacterially related index, coupled with CAL being an index representing the destruction of periodontal tissues caused by inflammation, it is plausible that the changes in BPIFA1 in T2DM subjects might be largely attributed to responses to a systemic inflammation. However, in NDM subjects, the changes in salivary BPIFA1 might be mainly attributed to local immune responses to bacterial infection. However, the specific mechanisms underlying these hypotheses require further exploration.

BPIFA1 can regulate the proinflammatory mediators and cytokines such as TNF-*α* and other interleukins (ILs) stimulated by the binding of LPS and innate immune receptors [13, 14, 40]. A recent study showed that BPIFA1 could modulate the inflammatory response through the regulation of the TLR9/NF-*κ*B signaling pathway, which might further stimulate the expression of IL-6, IL-8, IL-1*β*, and TNF-*α in vitro* [26] but there have been no studies in saliva. Consequently, we detected levels of expressed TNF-*α* and IL-6 in saliva. We found that among NDM individuals, the concentration of salivary TNF-*α* displayed a similar trend to salivary BPIFA1. BPIFA1 might also exert an anti-inflammatory effect by regulating the expression of salivary TNF-*α*. However, among T2DM subjects, the changing trend in salivary TNF-*α* concentration was not consistent with that of salivary BPIFA1, indicating that the systemic inflammatory status caused by T2DM might also be influenced by other factors. However, the changing trend in salivary IL-6 concentration was not similar to that of salivary BPIFA1. One explanation might be that salivary IL-6 is not regulated by salivary BPIFA1. Another explanation might be due to the low expression level of IL-6 in saliva; we could not accurately detect this trend. To evaluate the relationship between T2DM and levels of BPIFA1, TNF-*α*, and IL-6 in saliva, logistic regression analysis was used and we found that none of these were independent risk indicators for T2DM.

## 5. Conclusions

We demonstrated that BPIFA1 is present at significant concentrations in saliva and can be used as a sensitive biomarker of T2DM, especially in patients with severe periodontitis and nonperiodontitis. Among NDM subjects, salivary BPIFA1 might exert an anti-inflammatory effect by regulating the expression of salivary TNF-*α*.

## Supplementary Material

Supplementary Fig. 1 A schematic diagram showing the antimicrobial and anti-inflammatory effects of BPI fold containing family A, member 1 (BPIFA1). BPIFA1 has antibacterial properties since it can bind bacterial lipopolysaccaride (LPS) and is likely to be bacteriostatic. Moreover, BPIFA1 could modulate the inflammatory response through the regulation of the Toll-like receptor (TLR) 9/NF-κB signaling pathway, which might further stimulate the expression of interleukin-6 (IL-6) and tumor necrosis factor-α (TNF-α). BPIFA1 can also directly regulate the proinflammatory cytokines such as IL-6 and TNF-α stimulated by the binding of LPS.

## Figures and Tables

**Table 1:** Description of clinical diagnostic criteria for plaque and bleeding indices.

Plaque index	0 = no plaque at the gingival margin.
	1 = a film of plaque adhering to the free gingival margin and adjacent area of the tooth. The plaque may only be recognized by running a probe across the tooth surface.
	2 = moderate accumulation of soft deposits within the gingival sulcus, on the gingival margin, and/or adjacent tooth surface, which can be seen by the naked eye.
	3 = abundance of soft matter within the gingival sulcus and/or at the gingival margin and adjacent tooth surface.

Bleeding index	0 = normal appearance of healthy gingiva.
	1 = color changes related to inflammation but no bleeding.
	2 = slight bleeding that remains at the point of sampling.
	3 = bleeding extending from the point of sampling and flowing around the gingival margin.
	4 = profuse bleeding that overflows the gingival margin.
	5 = spontaneous bleeding.

**Table 2:** Characteristics of individuals with (patients) and without (healthy) type 2 diabetes.

	NDM	T2DM	*P* value
	*N* = 44	*N* = 44	
Age (in years, mean ± SD)	53.7 ± 9.2	58.0 ± 10.4	0.599^a^
Nonelderly (<60), % (*n*)	68% (30)	61% (27)	0.656^b^
Elderly (≥60), % (*n*)	32% (14)	39% (17)	
Gender, % (*n*)			
Males	75% (33)	54.5% (24)	0.073^b^
Females	25% (11)	45.5% (20)	
FBG (mg/dL, mean ± SD)	8.37 ± 2.62	5.06 ± 0.53	<0.001^a^
Normal FBG (<6.1 mg/dL), % (*n*)	95% (42)	18% (8)	<0.001^b^
Elevated FBG (≥6.1 mg/dL), % (*n*)	5% (2)	82% (36)	
BMI (mean ± SD)	25.85 ± 2.98	24.38 ± 2.51	0.208^a^
Nonobese (BMI ≤ 28), % (*n*)	86% (38)	84% (37)	1.000^b^
Obese (BMI > 28), % (*n*)	14% (6)	16% (7)	
Number of teeth (mean ± SD)	27.61 ± 1.35	27.86 ± 1.63	0.389^a^
Severity of periodontitis, % (*n*)			
No periodontitis	25% (11)	25% (11)	1.000^b^
Mild	25% (11)	25% (11)	
Medium	25% (11)	25% (11)	
Severe	25% (11)	25% (11)	

T2DM: type 2 diabetes mellitus; NDM: nondiabetes mellitus; SD: standard deviation; BMI: body mass index; FBG: fasting blood glucose. ^a^Independent sample *t*-test. ^b^Chi-squared test.

**Table 3:** Periodontal parameters of various severities of periodontitis with or without T2DM.

	NDM				T2DM				*P* value
	No periodontitis (G1)	Mild periodontitis (G2)	Moderate periodontitis (G3)	Severe periodontitis (G4)	No periodontitis (G5)	Mild periodontitis (G6)	Moderate periodontitis (G7)	Severe periodontitis (G8)	
PPD (median (IQR))	2.00 (2.00–2.00)	2.57 (2.57–2.57)	2.57 (2.61–2.39)	2.79 (3.21–2.00)	2.00 (2.00–2.00)	2.57 (2.57–2.57)	2.11 (2.36–2.00)	2.50 (2.79–2.21)	*P*_1_ = 0.847^b^
									*P*_2_ < 0.01^a^
									*P*_3_ = 0.01^a^

CAL (median (IQR))	0.00 (0.00–0.00)	0.75 (1.07–0.75)	1.07 (1.21–0.71)	1.79 (2.14–1.54)	0.00 (0.00–0.00)	0.75 (1.07–0.75)	0.71 (1.46–0.57)	1.41 (1.79–0.93)	*P*_1_ = 0.428^b^
									*P*_2_ < 0.01^a^
									*P*_3_ < 0.01^a^

BI (median (IQR))	0.00 (0.00–0.00)	0.79 (0.79–0.79)	1.43 (1.68–1.36)	1.96 (2.29–1.82)	0.00 (0.15–0.00)	0.79 (0.95–0.79)	1.47 (2.36–1.29)	1.50 (2.04–1.29)	*P*_1_ = 0.768^b^
									*P*_2_ < 0.01^a^
									*P*_3_ < 0.01^a^

PLI (median (IQR))	0.86 (0.86–0.86)	2.04 (2.04–2.04)	2.29 (2.29–2.29)	2.75 (2.75–2.75)	0.86 (2.00–0.86)	2.04 (2.04–2.04)	2.29 (2.32–2.04)	2.75 (2.75–1.33)	*P*_1_ = 0.296^b^
									*P*_2_ = 0.02^a^
									*P*_3_ < 0.01^a^

T2DM: type 2 diabetes mellitus; NDM: nondiabetes mellitus; PPD: probing pocket depth; CAL: clinical attachment loss; PLI: plaque index; BI: bleeding index; IQR: interquartile range. *P*_1_: T2DM versus NDM; *P*_2_: within NDM group comparison; *P*_3_: within T2DM group comparison. ^a^Kruskal-Wallis between T2DM and NDM group; ^b^Mann–Whitney comparison of T2DM or NDM group.

**Table 4:** Salivary BPIFA1 (*μ*g/mL, median (IQR)), TNF-*α*, and IL-6 (pg/mL, median (IQR)) concentrations according to age, gender, fasting blood glucose, and BMI.

	BPIFA1 (median (IQR))		TNF-*α* (median (IQR))		IL-6 (median (IQR))	
	NDM	T2DM	NDM	T2DM	NDM	T2DM
Nonelderly (<60)	629.15 (1540.31–265.34)	236.28 (1316.11–103.48)	210.60 (293.03–67.12)	157.56 (455.64–58.23)	8.20 (14.00–3.21)	11.40 (28.02–2.61)
Elderly (≥60)	727.16 (1780.33–167.20)	280.79 (588.73–144.02)	67.16 (558.48–25.84)	110.35 (390.03–68.20)	7.84 (13.80–5.35)	12.22 (33.57–4.52)
*P* value	*P*_1_ = 0.804^a^	*P*_2_ = 0.771^a^	*P*_1_ = 0.459^a^	*P*_2_ = 0.825^a^	*P*_1_ = 0.881^a^	*P*_2_ = 0.739^a^
Males	607.65 (1488.18–261.78)	285.94 (1010.52–101.88)	253.77 (463.93–62.68)	116.44 (328.92–49.38)	5.47 (15.27–2.30)	11.40 (33.57–2.95)
Females	669.97 (2016.03–243.15)	226.88 (423.01–103.47)	97.17 (241.18–37.73)	264.34 (759.63–110.35)	9.44 (13.11–5.94)	13.91 (23.43–4.20)
*P* value	*P*_1_ = 0.917^a^	*P*_2_ = 0.586^a^	*P*_1_ = 0.194^a^	*P*_2_ = 0.033^a^	*P*_1_ = 0.569^a^	*P*_2_ = 0.597^a^
Normal fasting blood glucose (<6.1 mg/dL)	630.17 (1600.61–243.77)	415.49 (2317.82–110.59)	158.68 (269.21–44.5)	127.74 (595.21–62.86)	8.11 (13.48–4.67)	4.27 (37.84–1.63)
Elevated fasting blood glucose (≥6.1 mg/dL)	939.59 (1503.59–375.59)	253.84 (875.29–100.94)	621.17 (988.54–253.79)	151.31 (393.48–60.22)	8.31 (14.77–1.84)	12.54 (29.81–4.43)
*P* value	*P*_1_ = 0.707^a^	*P*_2_ = 0.742^a^	*P*_1_ = 0.248^a^	*P*_2_ = 0.756^a^	*P*_1_ = 0.784^a^	*P*_2_ = 0.225^a^
Nonobese (BMI ≤ 28)	644.57 (1635.69–348.47)	285.94 (892.04–103.61)	189.70 (296.21–44.5)	145.12 (361.77–59.25)	8.75 (13.75–4.67)	10.00 (29.21–2.95)
Obese (BMI > 28)	405.00 (765.98–99.65)	201.29 (1598.02–82.00)	146.09 (733.95–46.26)	211.64 (1190.07–58.23)	6.41 (13.33–3.14)	12.86 (72.09–1140)
*P* value	*P*_1_ = 0.123^a^	*P*_2_ = 0.731^a^	*P*_1_ = 0.838^a^	*P*_2_ = 0.294^a^	*P*_1_ = 0.613^a^	*P*_2_ = 0.090^a^

T2DM: type 2 diabetes mellitus; NDM: nondiabetes mellitus; BPIFA1: BPI fold containing family A, member 1; TNF-*α*: tumor necrosis factor-*α*; IL-6: interleukin-6; BMI: body mass index; IQR: interquartile range.

*P*_1_: comparison of NDM; *P*_2_: comparison of T2DM. ^a^Independent sample *t*-test.

**Table 5:** Salivary BPIFA1 (*μ*g/mL, median (IQR)), TNF-*α*, and IL-6 (pg/mL, median (IQR)) concentrations according to severity of periodontitis with or without T2DM.

	NDM				T2DM				*P* value
	No periodontitis (G1)	Mild periodontitis (G2)	Moderate periodontitis (G3)	Severe periodontitis (G4)	No periodontitis (G5)	Mild periodontitis (G6)	Moderate periodontitis (G7)	Severe periodontitis (G8)	
BPIFA1 (pg/mL) (median (IQR))	879.89 (1592.79–550.00)	626.02 (713.92–387.36)	195.08 (877.94–74.76)	1441.96 (2342.94–375.59)	110.00 (285.94–82.00)	811.28 (1316.11–115.28)	573.87 (1963.67–236.28)	188.05 (340.13–55.81)	*P*_1,3_ = 0.019^a^; *P*_3,4_ = 0.024^a^
									*P*_5,6_ = 0.042^a^; *P*_5,7_ = 0.005^a^
									*P*_6,8_ = 0.021^a^; *P*_7,8_ = 0.002^a^
									*P*_1,5_ = 0.001^a^; *P*_4,8_ = 0.001^a^

TNF-*α* (pg/mL) (median (IQR))	360.42 (715.93–213.74)	96.16 (270.72–10.42)	58.25 (188.67–10.38)	253.79 (364.73–38.68)	118.63 (637.59–46.44)	116.44 (425.19–50.36)	201.20 (580.28–110.35)	84.07 (228.44–19.62)	*P*_1,2_ = 0.002^a^; *P*_1,3_ < 0.001^a^
									*P*_3,4_ = 0.036^a^; *P*_7,8_ = 0.042^a^
									*P*_3,7_ = 0.004^a^

IL-6 (pg/mL) (median (IQR))	3.74 (15.43–1.37)	6.33 (9.61–4.98)	9.45 (13.48–7.01)	8.66 (44.56–5.47)	10.00 (36.73–6.31)	18.15 (38.15–2.61)	15.89 (42.80–11.40)	3.89 (5.31–2.21)	*P*_7,8_ = 0.020^a^

T2DM: type 2 diabetes mellitus; NDM: nondiabetes mellitus; BPIFA1: BPI fold containing family A, member 1; TNF-*α*: tumor necrosis factor-*α*; IL-6: interleukin-6; BMI: body mass index. *P*_1,2_: G1 versus G2; *P*_1,3_: G1 versus G3; *P*_1,5_: G1 versus G5; *P*_3,4_: G3 versus G4; *P*_5,6_: G5 versus G6; *P*_5,7_: G5 versus G7; *P*_6,8_: G6 versus G8; *P*_7,8_: G7 versus G8; *P*_4,8_: G4 versus G8; *P*_3,7_: G3 versus G7. ^a^Analysis of variance (ANOVA).

**Table 6:** Logistic regression analysis of salivary BPIFA1, TNF-*α*, and IL-6 in subjects with or without T2DM.

		*P*	OR	95% CI	
				Lower limit	Upper limit
BPIFA1	1: 0–499 (pg/mL)	0.071	0.767	0.575	1.023
	2: 500–999 (pg/mL)				
	3: 1000–1499 (pg/mL)				
	4: 1500–1999 (pg/mL)				
	5: 2000–2499 (pg/mL)				
	6: 2500–2999 (pg/mL)				
	7: 3000–3500 (pg/mL)				

TNF-*α*	1: 0–199 (pg/mL)	0.611	0.893	0.577	1.382
	2: 200–399 (pg/mL)				
	3: 400–599 (pg/mL)				
	4: >600 (pg/mL)				

IL-6	1: 0–19 (pg/mL)	0.235	1.374	0.813	2.322
	2: 20–39 (pg/mL)				
	3: 40–59 (pg/mL)				
	4: >60 (pg/mL)				

Constant		0.610	1.364		

BPIFA1: BPI fold containing family A, member 1; TNF-*α*: tumor necrosis factor-*α*; IL-6: interleukin-6; OR: odds ratio; CI: confidence interval.
