# Supplementary material for: Associations of Salivary BPIFA1 Protein in Chronic Periodontitis Patients with Type 2 Diabetes Mellitus
Source: Int J Endocrinol. 2017 Oct 4;2017:1087017. doi: 10.1155/2017/1087017 (PMC5646319; doi:10.1155/2017/1087017)
Supplement: Supplementary file 1 — Supplementary Fig. 1 A schematic diagram showing the antimicrobial and anti-inflammatory effects of BPI fold containing family A, member 1 (BPIFA1). BPIFA1 has antibacterial properties since it can bind bacterial lipopolysaccaride (LPS) and is likely to be bacteriostatic. Moreover, BPIFA1 could modulate the inflammatory response through the regulation of the Toll-like receptor (TLR) 9/NF-κB signaling pathway, which might further stimulate the expression of interleukin-6 (IL-6) and tumor necrosis factor-α (TNF-α). BPIFA1 can also directly regulate the proinflammatory cytokines such as IL-6 and TNF-α stimulated by the binding of LPS. [file 1087017.f1.ppt]

## Slide 1
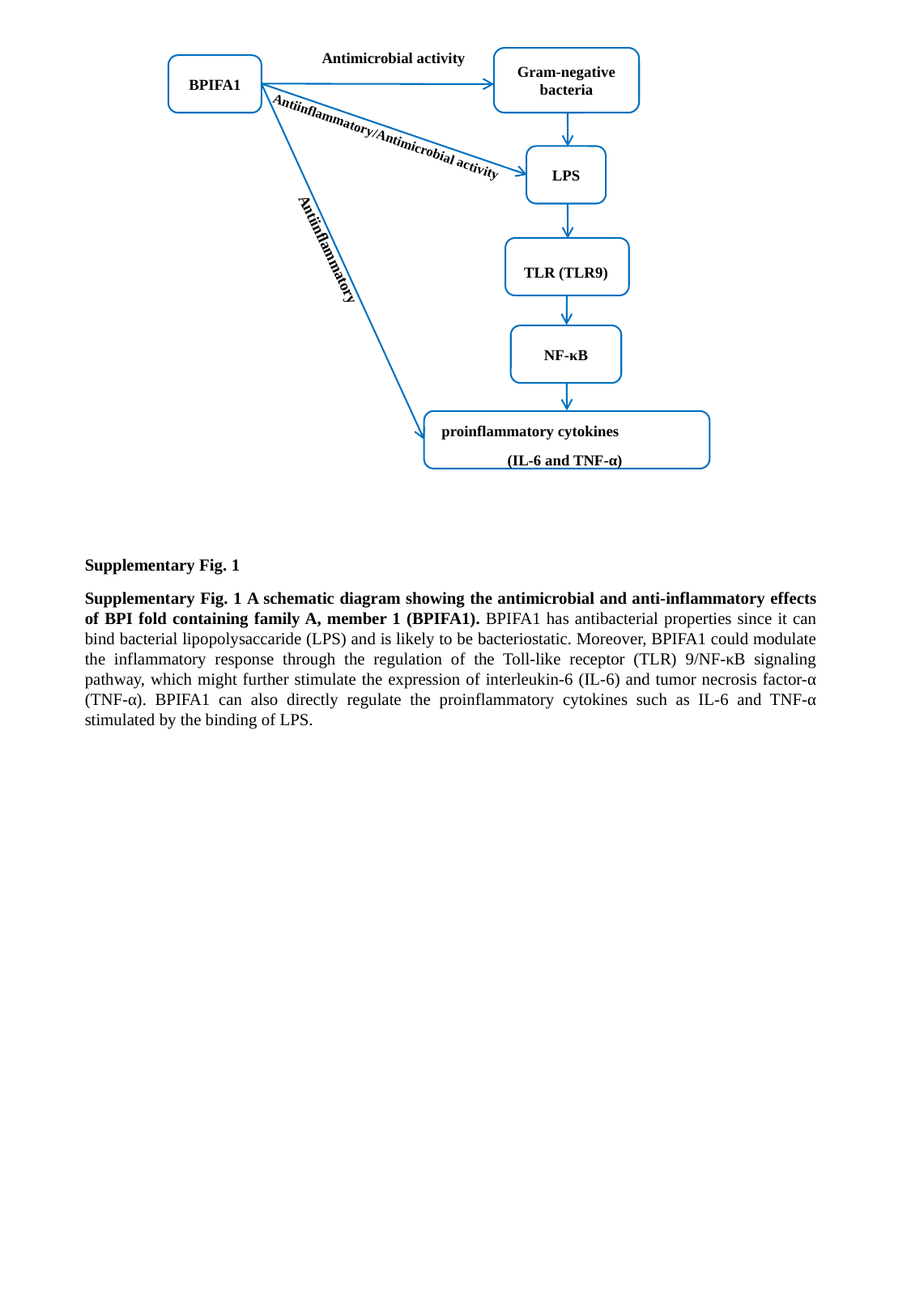

Antimicrobial activity
Gram-negative bacteria
BPIFA1
Antiinflammatory/Antimicrobial activity
LPS
Antiinflammatory
 TLR (TLR9)
NF-κB
proinflammatory cytokines (IL-6 and TNF-α)
Supplementary Fig. 1
Supplementary Fig. 1 A schematic diagram showing the antimicrobial and anti-inflammatory effects of BPI fold containing family A, member 1 (BPIFA1). BPIFA1 has antibacterial properties since it can bind bacterial lipopolysaccaride (LPS) and is likely to be bacteriostatic. Moreover, BPIFA1 could modulate the inflammatory response through the regulation of the Toll-like receptor (TLR) 9/NF-κB signaling pathway, which might further stimulate the expression of interleukin-6 (IL-6) and tumor necrosis factor-α (TNF-α). BPIFA1 can also directly regulate the proinflammatory cytokines such as IL-6 and TNF-α stimulated by the binding of LPS.
